# Comparison of Cancer Survival Trends in Hungary in the Periods 2001–2005 and 2011–2015 According to a Population-Based Cancer Registry

**DOI:** 10.3389/pore.2022.1610668

**Published:** 2022-09-06

**Authors:** István Kenessey, Georgina Szőke, Mária Dobozi, István Szatmári, András Wéber, György Fogarassy, Péter Nagy, Miklós Kásler, Csaba Polgár, Ágnes Vathy-Fogarassy

**Affiliations:** ^1^ National Institute of Oncology, Budapest, Hungary; ^2^ National Tumor Laboratory Project, Budapest, Hungary; ^3^ Department of Pathology, Forensic and Insurance Medicine, Semmelweis University, Budapest, Hungary; ^4^ Department of Computer Science and Systems Technology, University of Pannonia, Veszprém, Hungary; ^5^ Cancer Surveillance Section, International Agency for Research on Cancer (IARC/WHO), Lyon, France; ^6^ 1st Department of Cardiology, State Hospital for Cardiology, Balatonfüred, Hungary; ^7^ Department of Anatomy and Histology, University of Veterinary Medicine, Budapest, Hungary; ^8^ Institute of Oncochemistry, University of Debrecen, Debrecen, Hungary; ^9^ Department of Oncology, Semmelweis University, Budapest, Hungary

**Keywords:** mortality, survival, incidence, oncology, cancer registry, trend

## Abstract

**Background:** Assessment of population-based cancer survival may provide the most valuable feedback about the effectiveness of oncological surveillance and treatment.

**Aims:** Based on the database of the Hungarian National Cancer Registry, standardized incidence rates of lung, breast, colorectal, prostate and cervical cancer were compared to standardized mortality data of the Hungarian Central Statistical Office in the period between 2001 and 2015. Then survival analysis was performed on cleansed database.

**Results:** The incidence of colorectal, breast and prostate cancer increased, while standardized rates of lung and cervical cancer declined. The survival of colorectal, breast and prostate cancer showed improvement. Contrarily, lung cancer exhibited a mild decline, while that of cervical cancer did not change significantly. In earlier stages survival was improved among almost every studied tumor type, while in advanced stages improvement was not observed. Comparison of stage distribution revealed that in the 2011–2015 period colorectal, breast and prostate cancer cases were diagnosed at earlier stages, while lung and cervical cancer patients were typically discovered at more advanced stages.

**Discussion:** The outcome of advanced cancer treatments is better in earlier stages, which highlighted the importance of screening network. However, growth of oncological treatment costs with longer patient survival imposes a constantly increasing burden on society.

## Introduction

In the developed world, cancer represents the second most serious epidemiological burden after cardiovascular diseases with estimated 19.3 million new cases and 10.0 million deaths in 2020 [1]. International comparisons usually confirm that Hungary is among countries with the highest incidence and mortality rates [2].

Evaluation of population-based cancer survival may provide the most valuable information about effectiveness of oncological treatment, and promotes the establishment of a well-functioning national oncology network [3]. In Hungary reporting of new cancer cases is regulated by the decree of Ministry of Human Capacities, which ordered data collection to the Hungarian National Cancer Registry operated by the National Institute of Oncology. Besides evaluation of incidence according to ICD-10 coding system (World Health Organization’s International Statistical Classification of Diseases and Related Health Problems 10th Revision), Cancer Registry collects additional patient follow-up data as well as tumor characteristic features (e.g., TNM stage, histology type) and details of the applied treatment modalities. In practice, healthcare providers filter data from their own hospital information systems quarterly, produce a report and submit it to the specialized website of the Cancer Registry. The base of registration is the unique health insurance ID, which allows connecting records from different reports and avoiding double registrations.

Based on death certificates, the Hungarian Central Statistical Office publishes annual mortality statistics; however, in the absence of previous medical data performance of dynamic follow-up analysis is not feasible. The National Health Insurance Fund of Hungary provides financial background of healthcare, which completely covers the domestic population. Excluding private healthcare providers, this environment allows the establishment of a relatively complete database of medical costs and recording of current survival status. Due to trilateral exchange, survival status of cancer patients is available for the Cancer Registry from distinct sources, which support data verification and completeness.

The available diagnostic machinery and applied treatments determine the efficacy of oncologic therapy. Advanced diagnostic tools and organization of screening network allow detecting cancer at earlier stages. On the other hand, during the past 2 decades revolutionary breakthrough discoveries led to paradigm shift in the treatment of oncologic patients. In addition to traditional surgery, chemotherapy and irradiation, the introduction of target-based therapy and later immunotherapies opened new horizons for cancer groups where previously limited options were available for interventions. Compared to the traditional methods, novel therapeutic modalities increased the costs of oncologic treatments drastically, therefore nowadays data about the efficacy of treatments is more important than ever before. The clearest marker of quality assurance is the evaluation of survival. Previously a large international study demonstrated population-based cancer survival, mortality, and incidence in seven high-income countries between 1995 and 2014 [3]. Contrarily, in the majority of countries only estimated cancer survival data are available, publication of population-based follow-up information is still limited [4].

The aim of our study was to evaluate cancer survival patterns in Hungary representing the malignancies with the highest public health risk (e.g., lung, breast, colorectal, prostate and cervical cancer). In addition, a statistical comparison of two different eras was performed to demonstrate survival trends. In the first period mostly traditional treatment modalities were applied, while later innovative therapeutic options were available, therefore, comparison provided an opportunity to evaluate the development of effectiveness in oncology. Since appropriateness of traditional and innovative therapeutic options depends on how advanced the malignant process is, our results were stratified according to cancer stage as well.

## Materials and Methods

### Data Source

Decree of Ministry of Human Capacities regulated the activity of population-based Hungarian National Cancer Registry according to the international guidelines since 1999. Cancer Registry covers the whole Hungarian population, and valid data are available from 2000. Data of new patients in the period between 2001 and 2015 were extracted from the database regarding type of diagnosed malignancy, date of discovery, medical visits, last follow-up, and last survival status. Primary selection of the enrolled population was based on ICD-10 coding system [5]. Patients were included in this study with received diagnosis of colorectal cancer (ICD-10: C18, C19, C20, C21), trachea and lung cancer (C33, C34), breast cancer (C50), cervical cancer (C53) or prostate cancer (C61). TNM parameters were also extracted from the database, coding of stage was based on UICC’s TNM Classification of Malignant Tumours, 8th edition, except for prostate cancer, where the 7th edition was applied [6, 7]. Cancer stage was considered without modification marks (e.g., stage IA and IB were also assessed as stage I).

Detailed demographic data and national descriptive mortality database were officially purchased from the Hungarian Central Statistical Office (Budapest, Hungary) [8].

### Age Standardization

To calculate age-standardized incidence (ASIR) and mortality rates (ASMR) for 100,000 persons, 5-year age groups were counted according to the European Standard Population (2013) [9].

### Data Cleansing

To increase the quality of statistical analysis, only reliable data were kept. Patients with incomplete personal data or wrong discovery or examination date were deleted from the database. To increase specificity of the study, only those patients were involved in further analysis where one primary cancer was present, persons associated with multiple distinct malignancies were excluded. During survival analysis only cases with interpretable follow-up period were incorporated, patients that died within 1 month from the initial diagnosis were not taken into account. Occurrence of false diagnoses was reduced by removing patients without at least three medical visits within 12 months or having no record over 30 days after initial diagnosis. During statistical analysis, first recorded TNM parameters were applied.

### Statistical Analysis

Morbidity and mortality trends were assessed by Spearman’s rank-order correlation. Overall survival analyses were done using the Kaplan-Meier method. Survival intervals were determined as the time period from discovery date to the time of death in months. The comparison between survival functions for different strata was assessed with the log-rank statistics. Distribution of cancer stage was analyzed by Chi-square test. Statistical significance was determined when *p* values were under 0.05. Data analyses were done using R version 3.6.3 (The R Project for Statistical Computing, Vienna, Austria) [10] and Python version 3.8.3.

## Results

### Standardized Data of Cancer Incidence and Mortality in Hungary

**FIGURE 1:**
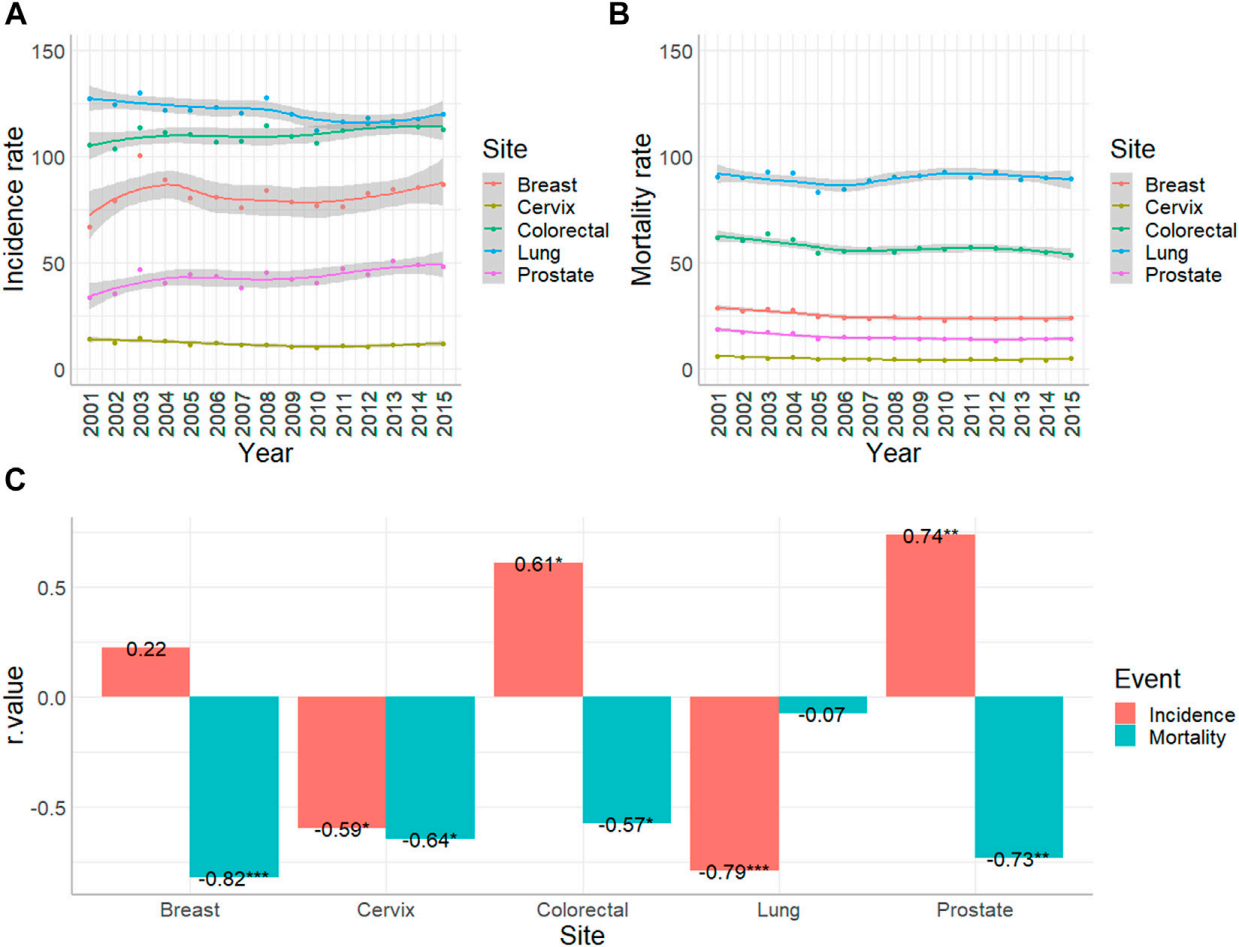
Age-standardized incidence and mortality rates of colorectal (C18-C21), lung and trachea (C33-34), breast (C50), cervical (C53) and prostate (C61) cancer per 100,000 people in Hungary between 2001 and 2015. **(A)** Standardized incidence rates with 95% confidence intervals were based on the data of Hungarian National Cancer Registry. **(B)** Standardized mortality rates with 95% confidence intervals were based on the data of Hungarian Central Statistical Office. **(C)** Result of Spearman’s rank order correlation to analyse trend changing (*: *p* < 0.05; **: *p* < 0.01; ***; *p* < 0.001).

Altogether, between 2001 and 2015 1,045,181 new cases were reported to the Cancer Registry with primary malignant tumor excluding non-melanocytic skin cancer. The database contained 147,748 colorectal, 168,279 lung, 112,739 breast, 17,282 cervical and 57,307 prostate cancer patients. Standardization for European Standard Population (2013) revealed that during the studied period lung cancer showed the highest incidence with an annual range of 112.4 and 130.3 cases per 100,000 persons. The standardized annual numbers of colorectal, breast, prostate and cervical cancer were between 103.6–116.7, 66.9–100.5, 33.8–51.2 and 10–14.5 cases per 100,000 persons, respectively (Figure 1A). At the same time Hungarian Central Statistical Office registered 484,640 deaths associated with malignant diseases. The standardized mortality of lung cancer proved to be the highest with an annual 83.4 to 93 cases per 100,000 capita. Colorectal, breast, prostate and cervical cancer proved to be in the range of 53.9–63.5, 23–28.6, 13.1–18.7 and 4.1–6 standardized cases per 100,000 persons per annum, respectively (Figure 1B).

Analyzing trends in the same era, Spearman rank order correlation proved that incidence of colorectal cancer increased (R = 0.61, *p* < 0.05), while mortality decreased (R = −0.57, *p* < 0.05). Incidence of lung cancer decreased (R = −0.79, *p* < 0.001), while mortality stagnated. Incidence of breast cancer did not show a significant change, while the number of deaths associated with breast cancers was reduced (R = −0.82, *p* < 0.001). During the analyzed period, standardized incidence and mortality rate of cervical cancer diminished (R = −0.59 and −0.64, respectively, *p* < 0.05). Incidence of prostate cancer showed an increase (R = 0.74, *p* < 0.01), while the mortality rate decreased (R = −0.73, *p* < 0.01) (Figure 1C).

### Survival Pattern

**FIGURE 2:**
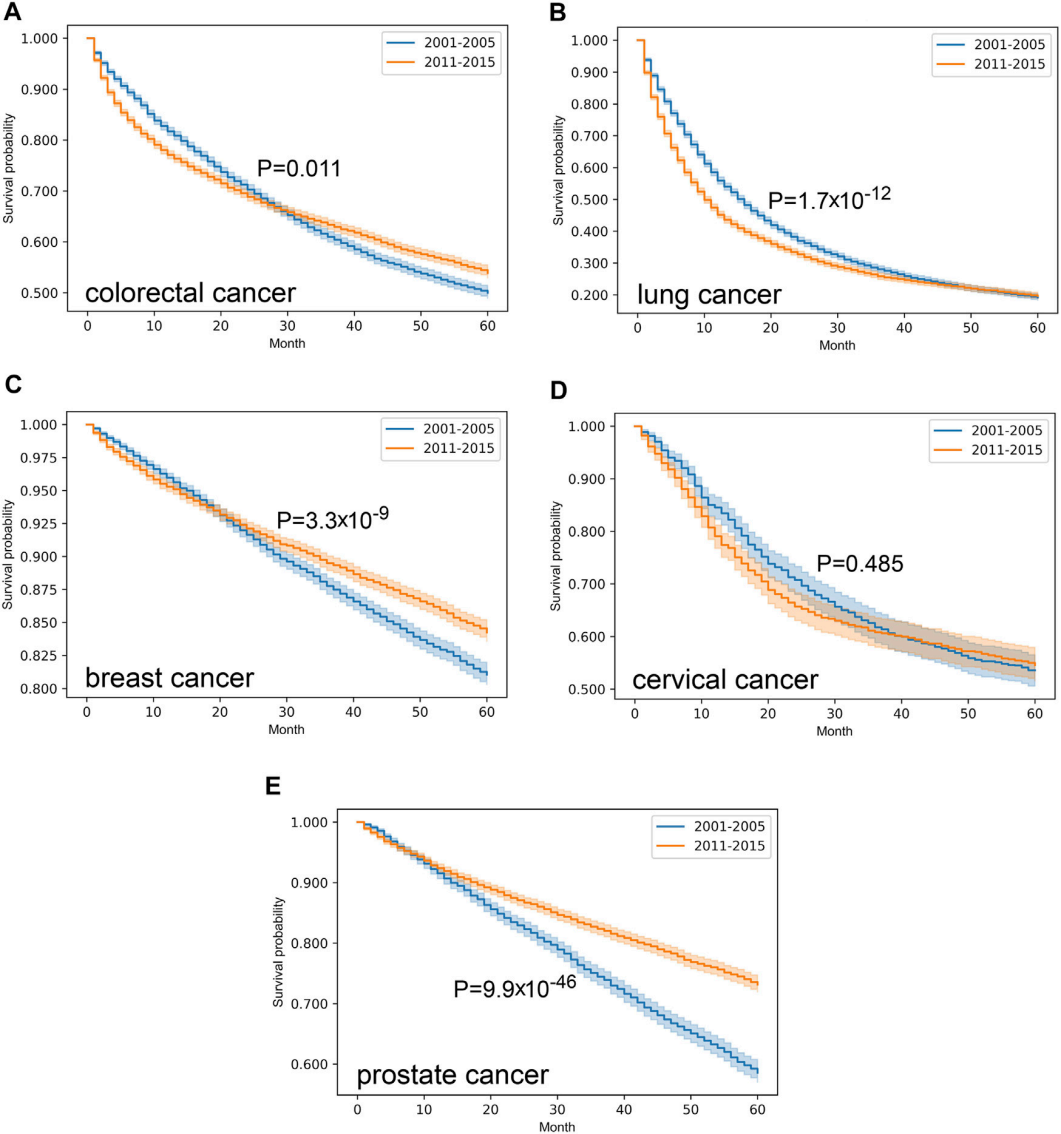
Overall survival with 95% confidence intervals of colorectal (C18–C21) **(A)**, lung (C33–C34) **(B)**, breast (C50) **(C)**, cervical (C53) **(D)** and prostate (C61) **(E)** cancer from 2001–2005 through 2011–2015 on cleansed population-based database of Hungarian National Cancer Registry.

In our survival analysis only those patients were involved, where complete datasets were available with considerable follow-up period, and only one primary malignancy was reported. Indeed, cases with late diagnosis or incidental findings were ruled out, as well as patients with multiple primary lesions. Application of the exclusion criteria made 9,016 and 12,182 colorectal, 7,919 and 8,570 lung, 11,787 and 13,784 breast, 1,257 and 1,545 cervical, 4,054 and 6,610 prostate cancer cases suitable for further analysis, regarding the periods of 2001–2005 and 2011–2015, respectively. Comparison of Kaplan-Meier curves in the two distinct eras revealed that colorectal (*p* = 0.011), breast (*p* = 3.3*10^–9^) and prostate cancer (*p* = 9.9*10^–46^) were associated with improved survival over time (Figure 2). Albeit the 5-year survival rate of lung cancer proved to be almost the same (approx. 20%), the fall of the curve was steeper in the later period that marks a more unfavorable outcome (*p* = 1.7*10^–12^). In contrast, survival of cervical cancer did not change (*p* = 0.485) from 2001–2005 to 2011–2015.

Survival data were stratified according to cancer stage, where this parameter was available. Analyzing of the distribution revealed that compared to 2001–2005, in 2011–2015 colorectal, breast and prostate cancer cases were discovered at earlier stages (*p* = 6.5*10^–27^, 1.6*10^–17^ and 1.3*10^–4^, respectively). On the other hand, lung (*p* = 1.6*10^–22^) and cervical cancer (*p* = 0.02) were diagnosed at a more advanced stage (Table 1). In case of colorectal cancer, the proportion of stage II cases increased, while that of stage III cases were reduced. Breast and prostate cancer showed an elevation in the proportion of stage I, and a decrease in that of stage II cases. Compared to the earlier period, the proportion of stage IV lung cancer patients increased, while in case of cervical cancer the majority of the new cases was discovered at stage III instead of stage I.

**TABLE 1:** Distribution of cancer Stages in the studied cancer types.

	2001–2005	2011–2015	*p*-value (Chi-square)
Colorectal			6.5 × 10^−27^
I	1700 (33.4%)	1777 (30.2%)	
II	958 (18.8%)	1608 (27.3%)	
III	1382 (27.2%)	1285 (21.8%)	
IV	1044 (20.5%)	1212 (20.6%)	
Lung			1.6 × 10^−22^
I	594 (10.7%)	646 (11.6%)	
II	1800 (32.4%)	1397 (25%)	
III	1851 (33.3%)	1842 (33%)	
IV	1306 (23.5%)	1700 (30.4%)	
Breast			1.6 × 10^−17^
I	2155 (36.5%)	2906 (44.3%)	
II	2543 (43.1%)	2403 (36.6%)	
III	582 (9.9%)	598 (9.1%)	
IV	624 (10.6%)	653 (10%)	
Cervix			0.02
I	241 (36.6%)	208 (31.6%)	
II	103 (15.6%)	91 (13.8%)	
III	181 (27.5%)	233 (35.4%)	
IV	134 (20.3%)	126 (19.1%)	
Prostate			1.3 × 10^−4^
I	616 (31.3%)	936 (36.5%)	
II	787 (40%)	914 (35.6%)	
III	139 (7.1%)	220 (8.6%)	
IV	425 (21.6%)	494 (19.3%)	

**FIGURE 3:**
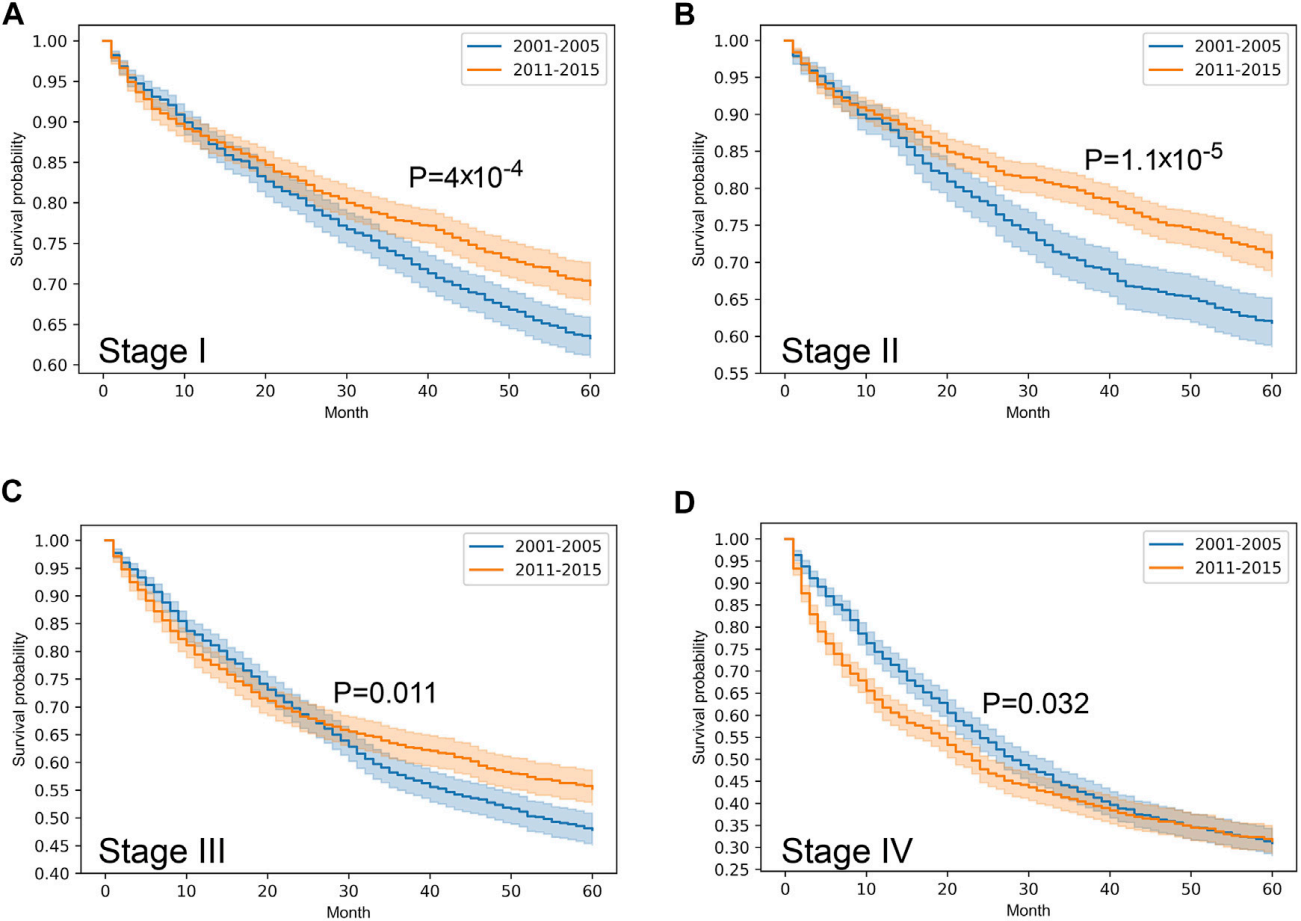
Overall survival with 95% confidence intervals of colorectal cancer (C18–C21) from 2001–2005 through 2011–2015 according to cancer stage. **(A)** stage I, **(B)** stage II, **(C)** stage III, **(D)** stage IV.

**FIGURE 4:**
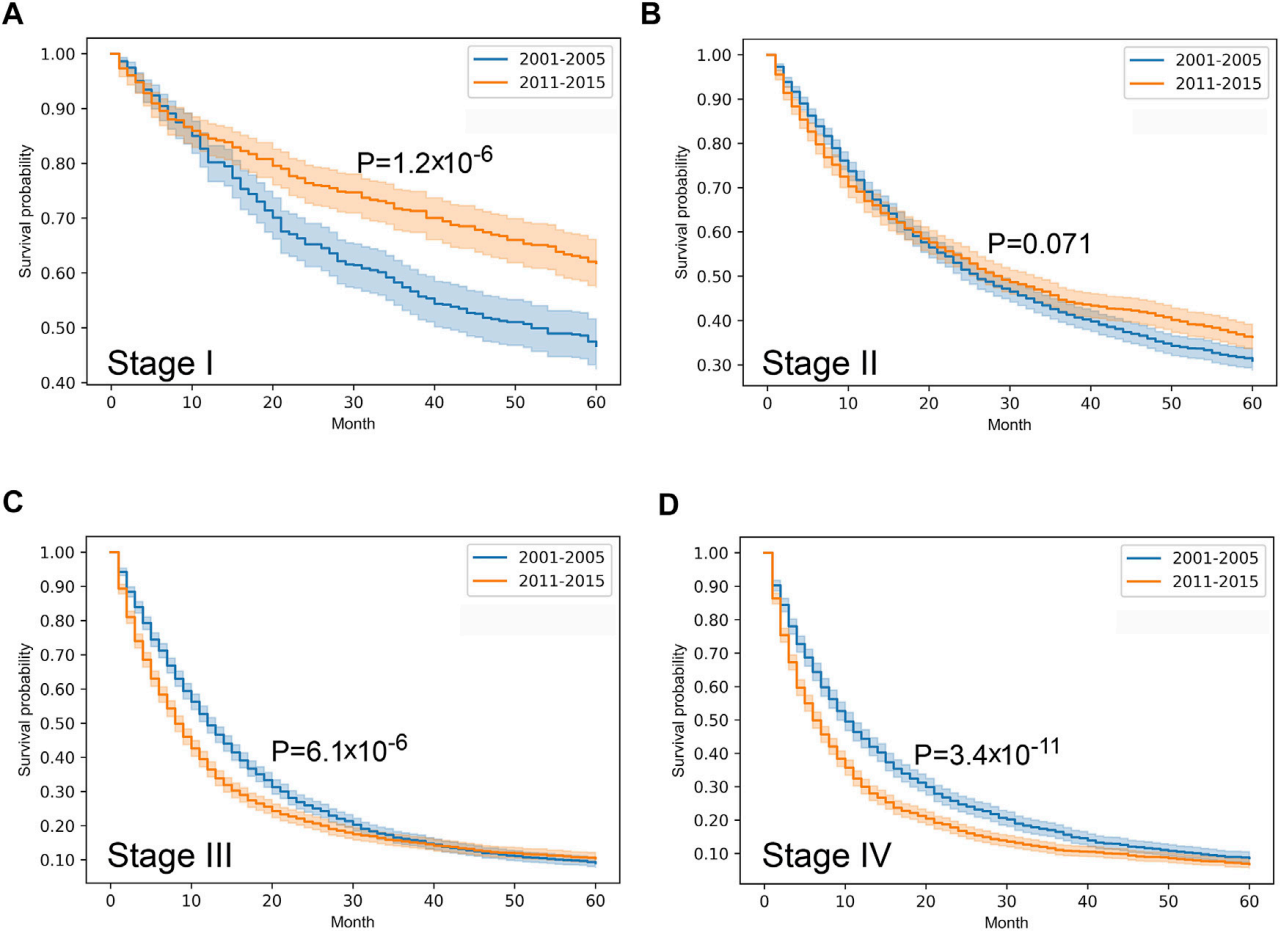
Overall survival with 95% confidence intervals of lung cancer (C33–C34) from 2001–2005 through 2011–2015 according to cancer stage. **(A)** stage I, **(B)** stage II, **(C)** stage III, **(D)** stage IV.

**FIGURE 5:**
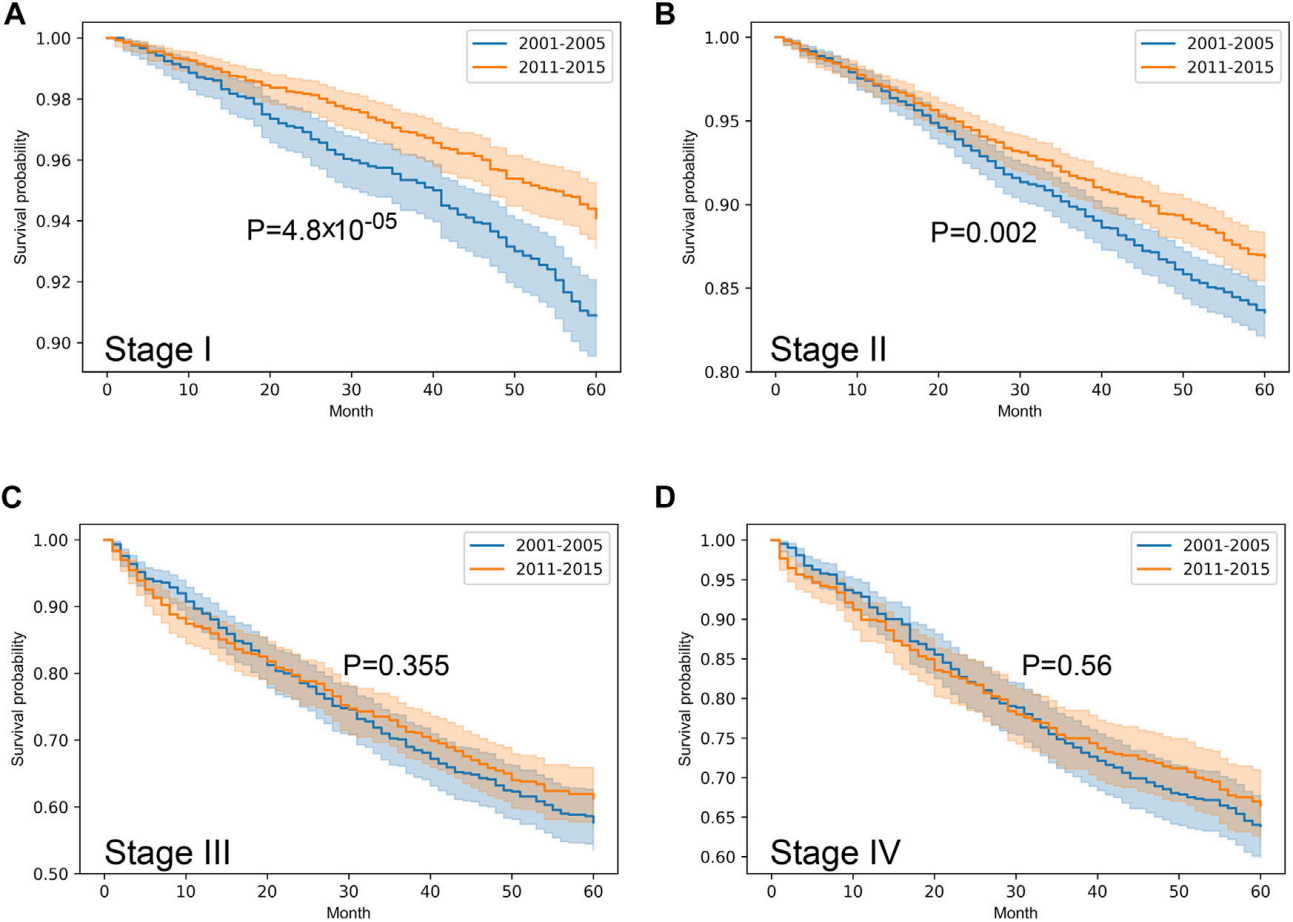
Overall survival with 95% confidence intervals of breast cancer (C50) from 2001–2005 through 2011–2015 according to cancer stage. **(A)** stage I, **(B)** stage II, **(C)** stage III, **(D)** stage IV.

**FIGURE 6:**
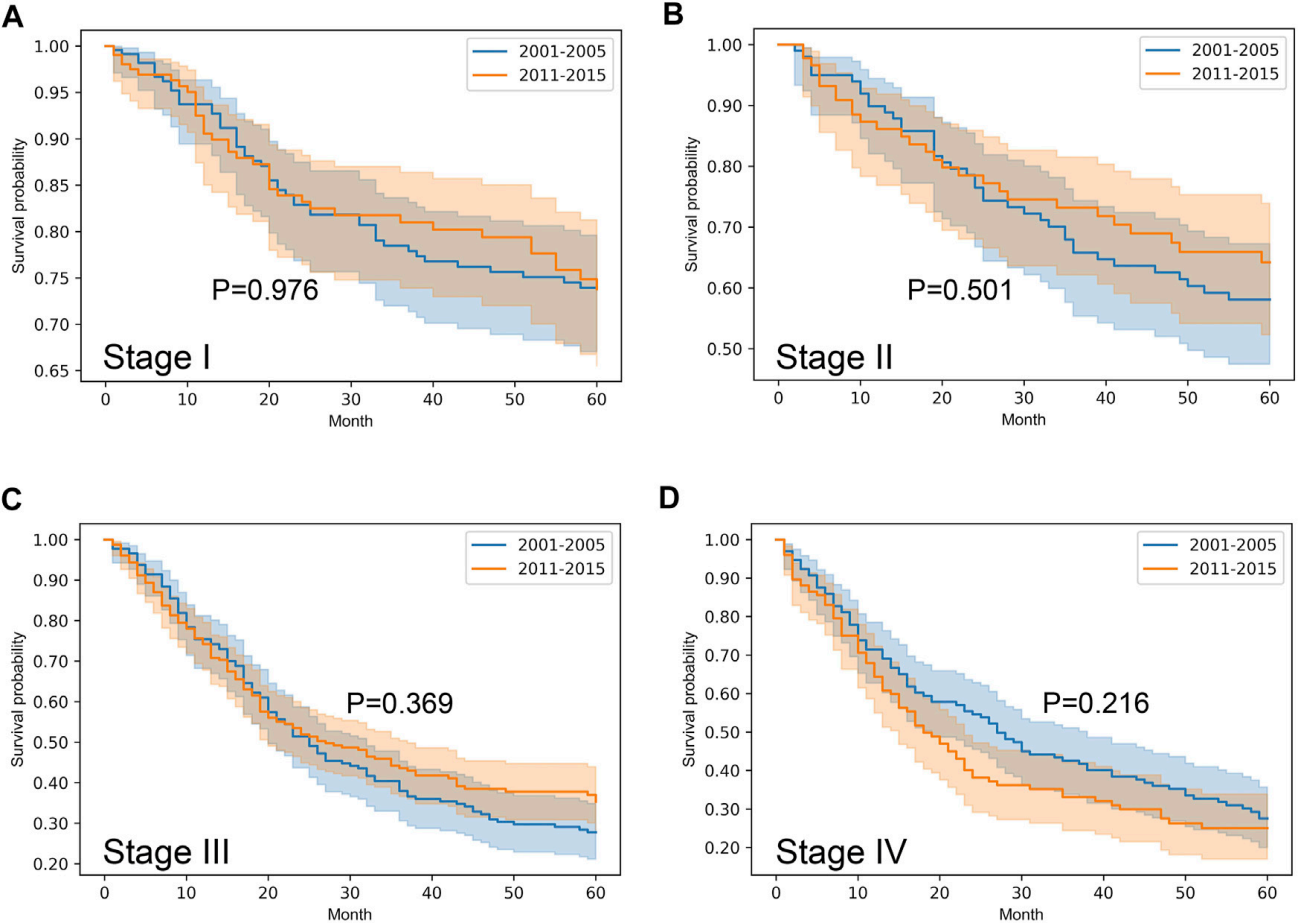
Overall survival with 95% confidence intervals of cervical cancer (C53) from 2001–2005 through 2011–2015 according to cancer stage. **(A)** stage I, **(B)** stage II, **(C)** stage III, **(D)** stage IV.

**FIGURE 7:**
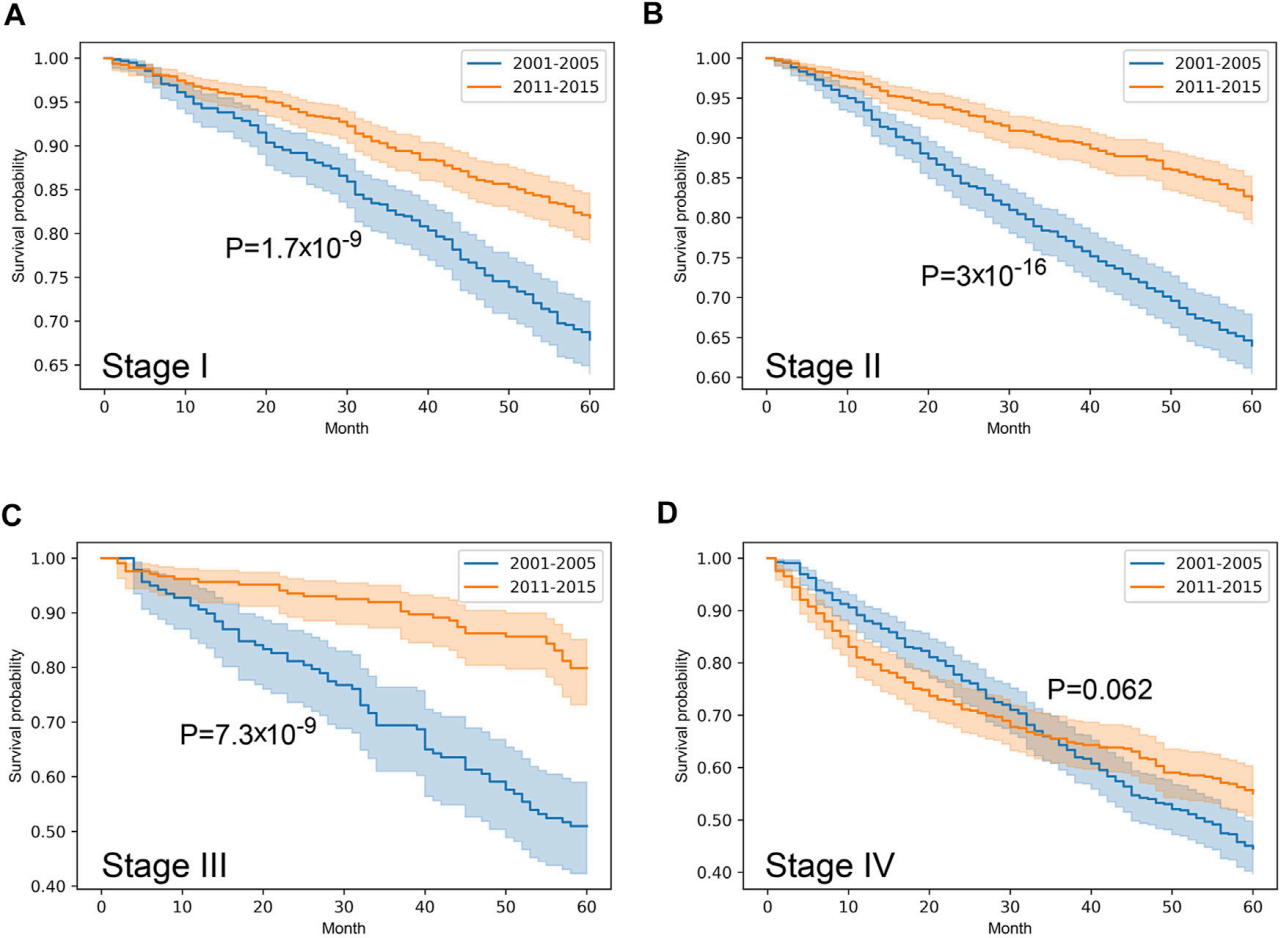
Overall survival with 95% confidence intervals of prostate cancer (C61) from 2001–2005 through 2011–2015 according to cancer stage. **(A)** stage I, **(B)** stage II, **(C)** stage III, **(D)** stage IV.

Stratified survival analysis proved that from 2001–2005 to 2011–2015 the outcome of colorectal cancer improved in stage I-III (*p* < 0.05), however, it deteriorated in stage IV cases (*p* = 0.03) (Figure 3). Stage I lung cancer cases were associated with better outcome in the later period (*p* = 1.2*10^–6^), stage II group did not show any difference, however, in stage III and IV cases survival became more unfavorable (*p* < 0.05) (Figure 4). In cases of the stage I and II breast cancer groups outcome has improved, while survival of stage III and IV cases did not show difference (Figure 5). Stratification of cervical cancer according to stage showed similar results that survival of the total patient group, since outcome did not differ in the two periods (Figure 6). From the studied tumor types, treatment of prostate cancer became the most successful, since survival of stage I-III groups excellently improved (*p* < 0.05), while outcome of stage IV cases did not change (*p* = 0.06) (Figure 7).

## Discussion

In the new millennium a revolutionary paradigm shift occurred in the field of oncology: beside the trinity of surgery-cytostatics-irradiation, modern treatment regimens shifted oncology interventions towards a more personalized direction [11]. According to the Hungarian National Institute of Pharmacy and Nutrition database, the first two targeted therapeutic agents were trastuzumab for Her2-positive breast cancer, and imatinib for bcr-abl-positive chronic myeloid leukaemia, which were approved in 2000 and 2001, respectively [12]. Thus, the new trend continued over the years: among others, target-based options appeared for special molecular types of non-small cell lung cancer (e.g. gefitinib), colorectal cancer (e.g., cetuximab), prostate cancer (e.g., leuprolide) and cervical cancer (e.g. bevacizumab). Nevertheless, the acceptance of novel medicines undoubtedly increased therapeutic costs compared to traditional modalities. Additionally, according to regular estimations, global cancer incidence and mortality show constant growth [2, 13, 14]. Furthermore, retrospective confirmatory studies are not available in the majority of cases, which poses numerous questions in relations to future directions of precision oncology [11].

During our population-based study survival pattern of five solid tumors was analyzed in two different periods: 2001–2005 is a relatively naïve era when targeted agents were not widely used in Hungary, and 2011–2015 when targeted and immune therapeutic agents were generally accepted for application. Based on the database of the Cancer Registry, comparison of 5-year survival between the periods of 2001–2005 and 2011–2015 revealed that colorectal cancer, breast cancer and prostate cancer were associated with better outcome. Prolonged survival proved to be consistent with epidemiological data, since standardized incidence of colorectal cancer, breast cancer and prostate cancer showed growth, while standardized mortality of those entities decreased between 2001 and 2015. These opposite trends in incidence and mortality was not present in case of cervical cancer, where both indicators declined, thus, survival did not change significantly. Interestingly, regarding the same tumor types, a US analysis revealed similar trends; however, this joint evaluation of the American Cancer Society (ACS), the Centers for Disease Control and Prevention (CDC), the National Cancer Institute (NCI), and the North American Association of Central Cancer Registries (NAACCR) covered a longer period [15]. In contrast to the survival outcome of the other four studied malignancies, in Hungary lung cancer showed a mild decline, which was in accordance with the measured reduction of incidence and unchanged mortality. US linked data of SEER registry and Medicare also confirmed that despite the usage of advanced medicines, a gain of 1.5 months in median survival of non-small cell lung cancer was observed from 2000 through 2011 [16]. During the analyzed period acute inpatient costs declined (from $29,376 to $23,731), whereas outpatient spending increased by 23% (from $37,931 to $46,642). Though these Hungarian data were not stratified by histological subtype, it does not seem to support revolutionary progress in the field of lung cancer care either.

On the other hand, cancer stage at discovery determines the expected therapeutic efficacy, since treatments are most effective in early detected malignancies, particularly prior to the onset of symptoms [17]. According to the evaluation of the American Cancer Society, in the US cancer death rates of 2015 dropped by 26% compared to that of 1990 [18]. This decrease proved to be more explicit in case of cancer sites where effective approaches for prevention and early detection were available: mortality of lung, colorectal, prostate and breast cancer decreased by almost 50%. In Denmark cancer became regarded as an acute life threatening disease, which accelerated the diagnostic process and treatment that prevent progression of the disease to an advanced form [3]. Our results also confirmed the rule of thumb: stage I-III colorectal, stage I lung, stage I-II breast and especially stage I-III prostate cancer showed longer survival in 2011–2015 than in 2001–2005, which also corroborates improved efficacy of oncology care. Surprisingly, survival of advanced cases with distant metastasis still remained at the same poor levels, which requires future investigations. Note that majority of innovative agents has been introduced in advanced stage, while in earlier stages traditional treatment options carry more weight. Regardless of cancer stage, survival of cervical cancer did not change. However, in contrast to the other studied tumor types, the therapeutic approach of cervical cancer did not show any revolution in the past 2 decades. Furthermore, our data suggest that despite widespread availability of precision medicine in the field of lung cancer, stage III and IV groups associated with a more unfavorable outcome in the later period. It is important to note that while usually colorectal, breast and prostate cancers were firstly diagnosed in earlier phase, lung and cervical cancer cases were typically discovered in more advanced stages in the later studied period than previously. This fact suggests the urgent improvement of lung and cervical cancer screening.

Due to its unequivocal advances, cancer immunotherapeutic agents become widespread in the past few years. Since among the studied tumor types these drugs were started to be introduced in Hungary in 2016, their potential effects only modestly contribute to the present data; however, it clearly should be the topic of future analyses.

In summary, our work suggests that taking into account the most common cancer types with the highest epidemiological risk, the standardized incidence of colorectal, breast and prostate cancer showed increase in the period of 2001 and 2015, while mortality showed a declining trend. Comparison of the period 2001–2005 and 2011–2015 revealed that the outcome of these tumor types were improved. However, the development of screening network could allow to discover patients in earlier stages, which would improve the expected prognosis even more. According to our analysis, the efficacy of secondary prevention may exceed the impact of novel therapeutics, since former may lead to earlier detection, when expected outcome is better, while latter affects patient groups that are more difficult to treat. Furthermore, longer survival and constant growth of oncological costs impose an elevated cancer burden on the society.

### Limitations of the Study

Patients recorded with multiple primary cancers were not involved in the survival analysis, however, incidence values contained all recorded cases. Assessable TNM status was available in approx. 50% of cases, all these parameters were reported by clinicians (cTNM). During survival analysis, classification of histological or molecular subtype was not applied. The present analysis did not incorporate treatment data, co-morbidities, age distribution and socio-economic conditions, the basis of differentiation was only the time of diagnosis.

## Data Availability

The raw data supporting the conclusion of this article will be made available by the authors, without undue reservation.
